# Polysaccharides from *Psoralea corylifolia* alleviate CTX-induced immunosuppression in mice by modulating gut microbiota- metabolite-immune signaling

**DOI:** 10.3389/fimmu.2026.1759122

**Published:** 2026-03-03

**Authors:** Zhenhua Yin, Guangyu Hong, Hao Zhang, Juanjuan Zhang, Qingfeng Guo, Kun Li, Li Wang, Lin Chen

**Affiliations:** 1 Henan Engineering Research Center of Chemistry and Biology of Medicinal Resources, Henan Comprehensive Utilization of Edible and Medicinal Plant Resources Engineering Technology Research Center, Zhengzhou Key Laboratory of Synthetic Biology of Natural Products, Zhengzhou Key Laboratory of Medicinal Resources Research, Henan Joint International Research Laboratory of Drug Discovery of Small Molecules, Huanghe Science and Technology College, Zhengzhou, China; 2 School of Pharmacy, Henan University, Kaifeng, China

**Keywords:** gut microbiota, immune signaling, immunoregulation, metabonomics, polysaccharides from *Psoralea corylifolia* L.

## Abstract

**Introduction:**

Although polysaccharides from *Psoralea corylifolia* L. (PPs) have been reported to possess immune-stimulatory effects, their precise mechanisms of action remain unclear.

**Methods:**

In this study, the potential mechanism of PPs in alleviating CTX-induced immunosuppression was investigated by analyzing the gut microbiota, metabolomics, and immune parameters in mice.

**Results:**

The results showed that PPs significantly alleviated CTX-induced immunosuppression, as evidenced by increased immune organ indices, improved intestinal mucosalintegrity, elevated serum levels of IL-6 and TNF-α, enhanced activities of ACP, LDH, SOD, and GSH-Px, and reduced MDA content. Western blot analysis indicated that PPs activated the NF-kB and MAPK signaling pathways and upregulated the expression of intestinal tight junction proteins (Claudin-1, Occludin, and ZO-1). Immunohistochemical results further revealed that PPs modulated the numbers of CD4^+^ and CD8^+^ T cells in the small intestine. Based on 16S rDNA sequencing and untargeted metabolomics analysis, PPs promoted the proliferation of Lachnospiraceae_NK4A136_group and *Lactobacillus*, while reducing the abundance of Prevotellaceae_UCG-001, f:Lachnospiraceae_Unclassified, and *Alloprevotella*, thereby ameliorating metabolic disorders and counteracting CTX-induced immunosuppression. Spearman’s correlation coefficient analysis indicated significant associations among gut microbiota, serum metabolites, and immune as well as antioxidant indicators.

**Discussion:**

These results suggested that PPs enhanced the immune response in immunocompromised mice by boosting antioxidant capacity, improving the intestinal barrier, modulating gut microbiota structure, and correcting metabolic disturbances.

## 1 Introduction

Immunosuppression is an abnormal immune state characterized by a diminished response to antigens, making individuals more susceptible to bacterial infections such as respiratory tract infections, urinary tract damage, sepsis, and meningitis (1). Patients often develop immunosuppression following HIV infection, organ transplantation, critical illness, or long-term immunosuppressive therapy (2). Although widely used immunosuppressants such as cyclophosphamide, levamisole and tacrolimus are clinically effective, they exhibit considerable toxicity and side effects, such as fever, neutropenia, leukopenia, allergic reactions, and hepatic or renal impairment (3, 4). Therefore, identifying alternative natural immunomodulators with high efficacy and low toxicity is of great significance for maintaining immune homeostasis.

Natural polysaccharides have attracted substantial attention due to their extensive immunomodulatory activities, low toxicity, biodegradability and good biocompatibility, making them promising natural immune enhancers (5). Numerous studies have shown that natural polysaccharides could modulate the functions of immune organs, regulate humoral and cellular immunity, influence cytokine secretion, and activate immune-related signaling pathways, thereby enhancing or restoring immune function (6, 7). These polysaccharides can stimulate pattern recognition receptors such as Toll-like receptors, activating macrophages to produce pro-inflammatory cytokines and chemokines, thereby coordinating innate and adaptive immune responses (8). Among these, the MAPK/NF-κB signaling pathways play a pivotal role in mediating immune responses, inflammation, and cellular survival, and it is critically involved in the pathophysiology of inflammatory, immune, tumor, and metabolic disorders (9).

In addition to directly modulating host immunity, natural polysaccharides can also indirectly influence immune activity by regulating the gut microbiota and their metabolites. A substantial body of research has confirmed that the gut microbiota and its metabolites (e.g., short-chain fatty acids, bile acids) serve as a critical link in maintaining host immune homeostasis (10, 11). Many polysaccharides are resistant to direct digestion and can reach the intestine intact, where they are fermented by microorganisms. This fermentation alters microbial composition, metabolite profiles, and intestinal morphology, thereby modulating immunity (12, 13). This suggests that the immunoregulatory effects of polysaccharides likely originate in the gut and are mediated by microbial fermentation and metabolic products. Because traditional microbiological techniques cannot fully capture these complex interactions, metabolomics provided a complementary tool that efficiently reflects the functional output of the microbiota, and reveals the biochemical and metabolic state of cells and tissues (14). Therefore, integrating 16S rDNA sequencing and non-targeted metabolomics provides a comprehensive strategy to elucidate the mechanisms of polysaccharides by “microbiota-metabolism-immunity” axis.

*Psoralea corylifolia* L., known in traditional Chinese medicine as “Buguzhi,” is the dried mature fruit of the plant. According to the *Notice of the Ministry of Health on Further Regulating the Management of Raw Materials for Health Foods*” (Wei Fa Jian Fa [2002] No. 51), *P. corylifolia* is approved for use in health foods. It contains various active compounds, including coumarins, terpenoids, phenols, and flavonoids, which exhibit multiple biological activities, including immunoregulatory potential (15). However, reports on the immunoregulatory activity of *P. corylifolia* polysaccharides, remain limited. Our research group has previously characterized the molecular weight, monosaccharide composition, and glycosidic linkages of polysaccharides isolated from *P. corylifolia* (16). We also demonstrated that these polysaccharides activate macrophages via the TLR4-mediated MAPK and NF-κB signaling pathways, upregulating protein mRNA expression and enhancing cytokine secretion in RAW264.7 cells (17). Furthermore, *in vitro* digestion and fermentation experiments revealed that *P. corylifolia* polysaccharides resisted hydrolysis in simulated saliva, gastric, and intestinal fluids but were fermented by mouse fecal microbiota, suggesting that their biological activities were largely mediated through microbial metabolism (18). Therefore, we speculate that PPs regulates the intestinal immune response through the modulation of gut microbiota and fecal metabolites, leading to enhanced immunity in immunosuppressed mice.

However, the mechanism underlying the interaction between *P. corylifolia* polysaccharides and intestinal microbiota remain unclear, especially their effects on serum metabolomics, gut flora composition, and the MAPK/NF-κB signaling pathways. Therefore, this study employed a cyclophosphamide-induced immunosuppressed mouse model to evaluate the effects of *P. corylifolia* polysaccharides on immune organ indices, humoral immunity, and oxidative stress. In addition, 16S rDNA high-throughput sequencing and Ultra-high performance liquid chromatography-tandem mass spectrometry system (UPLC-MS/MS) non-targeted metabolomics were integrated to identify differential intestinal flora and key metabolites. Spearman’s correlation analysis was used to construct a network linking microbiota, metabolites, and immune indicators, thereby systematically elucidating the mechanisms by which *P. corylifolia* polysaccharides alleviate immunosuppression from a multi-omics perspective. This study provides a foundation for developing *P. corylifolia* polysaccharides as potential prebiotics and for designing precision nutrition strategies.

## 2 Materials and methods

### 2.1 Chemicals and reagents

Cyclophosphamide (CTX) was purchased from Sigma-Aldrich (USA). Enzyme-linked immunosorbent assay (ELISA) kits for cytokines, including interleukin 6 (IL-6) and tumor necrosis factor *α* (TNF-*α*), were obtained from Beijing 4A Biotech Co., Ltd. (Beijing, China). The following assay kits were purchased from Nanjing Jiancheng Bioengineering Institute (Nanjing, China), including lactate dehydrogenase (LDH), acid phosphatase (ACP), superoxide dismutase (SOD), malondialdehyde (MDA), and glutathione peroxidase (GSH-Px). Primary antibodies against anti-phospho-c-Jun N-terminal kinase (p-JNK, mouse monoclonal, 1:500), JNK (rabbit monoclonal, 1:1000), anti-phospho-p38 (p-p38, rabbit monoclonal, 1:1000), p38 (rabbit monoclonal, 1:1000), anti-phospho-ERK (p-ERK, rabbit monoclonal, 1:1000), ERK (mouse monoclonal, 1:500), anti-phospho-NF-kappa-B p65 (p-p65, rabbit monoclonal, 1:500), p-65 (rabbit monoclonal, 1:500), *β*-actin (anti-rabbit antibody, 1:3000)), Zonula occludens-1 (rabbit monoclonal, 1:1000), Claudin-1 (rabbit monoclonal, 1:500) and Occludin (rabbit monoclonal, 1:500) were obtained from Wuhan Servicebio Biotechnology Co., Ltd. (Wuhan, China). Horseradish peroxidase (HRP)-conjugated secondary antibodies were used, including sheep anti-rabbit (1:3000) and sheep anti-mouse (1:1000). All other conventional chemicals were of the analytical grade.

### 2.2 Preparation of PPs

According to the previous extraction methods of our research group (16, 18), polysaccharides from *P. corylifolia* were extracted using hot water extraction followed by ethanol precipitation. Proteins were removed by Sevage method, and the resulting extract was freeze-dried to obtain purified polysaccharides PPs, which was used in this study. The main active fraction of PPs was PCp-I, with molecular weight of 2.721×10^4^. PCp-I mainly contained galactose, arabinose, mannose, xylose, rhamnose and glucose. Its main backbone consisted of →5)-*α*-Ara*f*-(1→, →2, 4)-*α*-Rha*p*-(→, →4)-*α*-Gal*p*-(1→ and *β*-Glc*p*-(1→ linkages. Detailed structural information was provided in our previous work (16).

### 2.3 Animal experiment design

**Figure 1:**
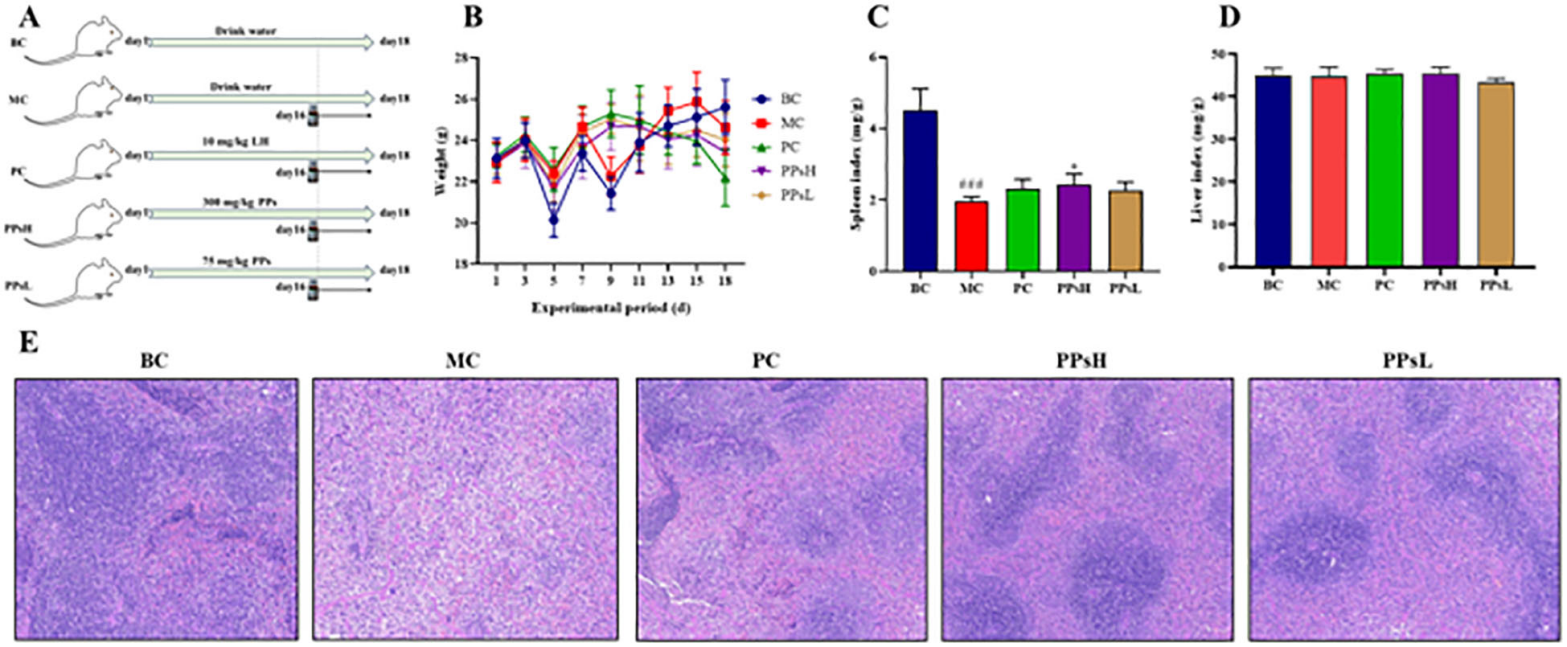
Experimental design and effect of PPs on body weight and immune organs in CTX-induced immunosuppressive mice. **(A)** Experimental design; **(B)** Body weight; **(C)** Spleen index; **(D)** Liver index; **(E)** Pathological observation of spleen by H&E staining (×200). ^###^*P* < 0.001 *vs*. BC. ^*^P < 0.05 *vs*. MC.

Male Balb/c mice (20 ± 2 g, 6–8 weeks old) from Zhengzhou Huaxing Animal Center (Henan) were housed under standard conditions (23-25 °C, 60–70% humidity, 12 h light/dark cycle) with free access to food and water at Huanghe Science and Technology College. All experiments complied with the ethical guidelines approved by the institution’s Academic Committee. Mice were randomly divided into five groups (n=12 per group) according to body weight. The experimental design was illustrated in **Figure 1A**. Blank control group (BC) and model control group (MC) were gavaged with 0.1 mL/10 g of normal saline, while positive control group (PC) received levamisole hydrochloride (LH, 10 mg/kg, 0.1 mL/10 g). The high-dose (HD) and low-dose (LD) groups were gavaged daily with PPs at 300 and 75 mg/kg, respectively, for 18 days. On days 16-18, mice in BC group were intraperitoneally injected with normal saline, whereas all other groups were injected with CTX (80 mg/kg/d) to induce immunosuppression. After fasting for 12 h, blood, spleen, liver, small intestine, and cecum contents were collected and stored at -80°C for subsequent analysis.

### 2.4 Body weight and immune organs indexes

Body weights were recorded every two days from the first day of gavage for a total of nine measurements. The spleen and liver were excised, rinsed with cold saline, blotted, and weighed. Organ indices were calculated according to the following Formula.


Spleen/Liver index=Spleen/Liver weight (mg)/Body weight (g)

### 2.5 Determination of Immune molecules and enzyme assay in serum

Blood samples were collected from the retro-orbital plexus, allowed to stand for 2 h at room temperature, centrifuged at 3500 r/min for 10 min to obtain serum. Serum levels of IL-6 and TNF-*α* were measured using ELISA kits. The activities of LDH and ACP were assayed using corresponding commercial kits according to the manufacturer’s instructions.

### 2.6 Detection of liver antioxidant activity

Liver tissues were accurately weighed and homogenized with 9 volumes of cold normal saline (weight-to-volume ratio = 1: 9) to prepare a 10% homogenate. After centrifugation at 3500 r/min for 10 min, the supernatant was collected. The activities of SOD and GSH-Px, as well as the MDA content were determined according to the instructions of the corresponding assay kits.

### 2.7 Pathological observation of spleen and intestinal tissue

Spleen and small intestine samples were fixed in 4% paraformaldehyde for at least 24 h, dehydrated, and embedded in paraffin. Paraffin blocks were sectioned into 3–4 μm slices using a microtome, deparaffinized, and rehydrated. Sections were stained with hematoxylin and eosin (H&E), dehydrated, cleared, and mounted with coverslips. Histological structures were observed using a bright-field microscope at 200× magnification.

### 2.8 Immunohistochemistry for intestinal CD4^+^ and CD8^+^ T cells

The distribution of CD4^+^ and CD8^+^ T cells in small intestinal tissue were assessed using immunohistochemical (IHC) staining. Paraffin-embedded tissue sections were deparaffinized, rehydrated, and subjected to antigen retrieval. Endogenous peroxidase activity was quenched with 3% hydrogen peroxide. Sections were encircled with an IHC pen and blocked with 3% bovine serum albumin (BSA). After washing with PBS, primary antibodies were applied and incubated overnight at 4°C. After washing three times with PBS, the sections were incubated with species-matched secondary antibodies, developed with DAB chromogen, and counterstained with hematoxylin. Finally, sections were dehydrated, mounted, and examined under a light microscope.

### 2.9 Western blot analysis

Spleen or small intestine tissues were rinsed with pre-cooled PBS, minced, and transferred to homogenization tubes containing 10 volumes of lysis buffer and two 4 mm homogenization beads. After homogenization, lysates were kept on ice for 30 min and centrifuged at 12,000 rpm for 10 min at 4°C. The supernatant was collected as the total protein extract. Protein concentration was measured using a BCA assay kit. Samples were mixed with 5×loading buffer (v/v= 4:1), boiled for 15 min, and stored at -20°C. Proteins were separated by SDS-PAGE (5% stacking gel, 10% separating gel) and transferred to PVDF membranes activated in methanol. Membranes were blocked with 5% skim milk for 30 min, incubated overnight at 4 °C with primary antibodies, washed three times with TBST, and incubated with HRP-conjugated secondary antibodies for 30 min. Protein bands were visualized using an enhanced chemiluminescence (ECL) reagent and imaged with an SCG-W3000 PLUS imaging system. Band intensities were quantified using ImageJ software.

### 2.10 16S rDNA sequencing of gut microbiota

Cecum contents were collected for genomic DNA extraction and quality assessment. Amplification of 16S rDNA variable region, library construction, high-throughput sequencing, and subsequent bioinformatics analysis were performed by Jinwei Zhi Biotechnology Co., Ltd. (Suzhou, China).

### 2.11 Non-targeted metabolomics analysis

Serum metabolites were analyzed using UPLC-MS/MS (Thermo Scientific Q Exactive HF). Raw data processed using Compound Discoverer 3.3 software (Thermo Fisher Scientific, USA) in combination with BMDB (BGI Metabolome Database), mzCloud database and ChemSpider online databases. Principal component analysis (PCA) and partial least squares discriminant analysis (PLS-DA) were used to evaluate metabolite variations among groups. Differential metabolites were screened based on variable importance in projection (VIP) ≥1, Fold Change ≥1.2 or ≤ 0.83, and *P* < 0.05. Metabolic pathways enrichment was conducted using the KEGG (http://www.genome.jp/kegg/) and HMDB (https://hmdb.ca/) databases. Spearman’s correlation analysis was performed to analyze associations between intestinal flora (genus level) and metabolites.

### 2.12 Statistical analysis

All statistical analyses were conducted using GraphPad Prism 8.0. Data were mean ± standard deviation (SD). Statistical differences among groups were determined using one-way analysis of variance (ANOVA). Differences were considered statistically significant at *P* < 0.05.

## 3 Results

### 3.1 Effect of PPs on the body weight and immune organs

PPs exerted positive effects on both body weight and immune organs in mice. The results are shown in **Figures 1B–D**. In the MC group, a significant decrease in body weight was observed three days after CTX injection (days 16-18). The body weight then remained consistently lower than that in the BC group. In contrast, mice in the PPsH and PPsL groups showed less weight reduction, suggesting that PPs alleviated CTX-induced weight loss. As shown in **Figures 1C, D**, CTX administration significantly reduced the spleen index compared with the BC group (*P* < 0.001), whereas both PPsH and PPsL treatment increased the spleen index in CTX-treated mice (*P* < 0.05). The effect on liver weight was minimal. These results indicated that PPs could mitigate CTX-induced immune organ damage.

Histological analysis of the spleen was performed (**Figure 1E**). In the MC group, CTX administration caused clear morphological damage. This included blurred boundaries between the red and white pulp, disorganized cellular structures, and a disruption of the normal tissue architecture. In contrast, PPs treatment restored clear medullary boundaries and dense cellular arrangements, suggesting effective protection of splenic structure from CTX-induced damage.

### 3.2 Effect of PPs on immune molecules and antioxidant activity

**Figure 2:**
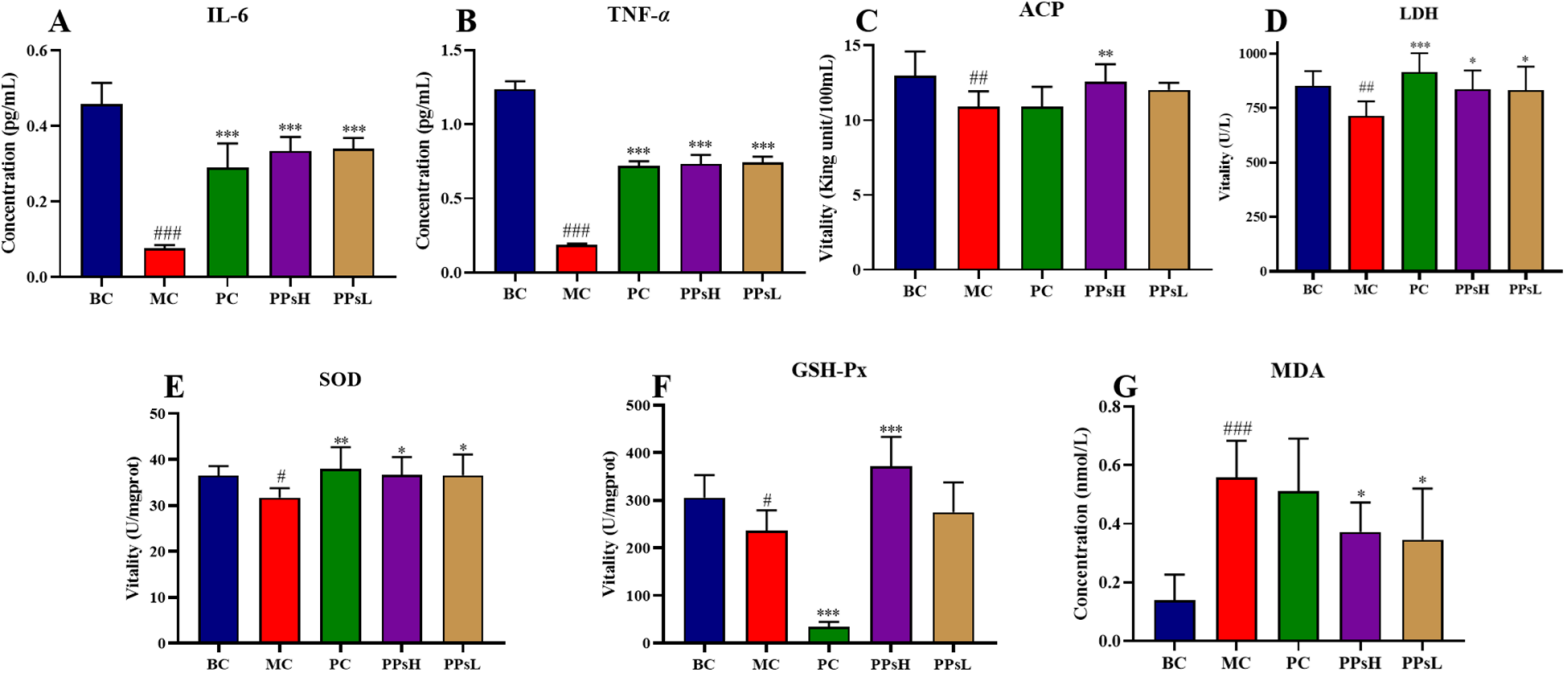
PPs enhanced inflammatory cytokine secretion, antioxidant capacity and immune activity in CTX-induced immunosuppressive mice. **(A)** Serum IL-6. **(B)** Serum TNF-*α*. **(C)** Serum ACP. **(D)** Serum LDH. **(E)** Liver SOD. **(F)** Liver GSH-Px. **(G)** Liver MDA. ^#^*P* < 0.05, ^##^*P* < 0.01, ^###^*P* < 0.001 *vs*. BC. ^*^P < 0.05, ^**^P < 0.01, ^***^P < 0.001 *vs*. MC.

IL-6 and TNF-*α* are key inflammatory markers in immune regulation. As shown in **Figures 2A, B**, CTX markedly reduced serum levels of both IL-6 and TNF-*α* compared with the BC group (*P* < 0.001), while both PPsH and PPsL significantly restored the concentrations of these cytokines (*P* < 0.001).

As shown in **Figures 2C, D**, the activities of ACP and LDH were significantly decreased in the MC group (*P* < 0.01). PPsH significantly increased the activities of both enzymes (*P* < 0.01 and *P* < 0.05). PPsL also produced a significant effect, notably elevating LDH activity (*P* < 0.05). These results indicate that PPs counteracted CTX-induced suppression of macrophage enzyme activity, thereby enhancing immune function.

The metabolism of CTX triggers excessive production of reactive oxygen species (ROS), leading to oxidative stress. This state was evidenced in our model by reduced activities of the key antioxidant enzymes SOD and GSH-Px, alongside elevated levels of the lipid peroxidation marker MDA in the liver. Both PPsH and PPsL significantly enhanced SOD and GSH-Px activities, reduced MDA content, and improved hepatic antioxidant capacity (**Figures 2E–G**). Collectively, these findings demonstrated the multifaceted protective effects of PPs. They alleviated CTX-induced suppression of cytokine secretion, enhanced hepatic antioxidant defenses, and thereby exerted overall anti-inflammatory effects.

### 3.3 Effect of PPs on NF-ĸB/MAPK signaling pathway

**Figure 3:**
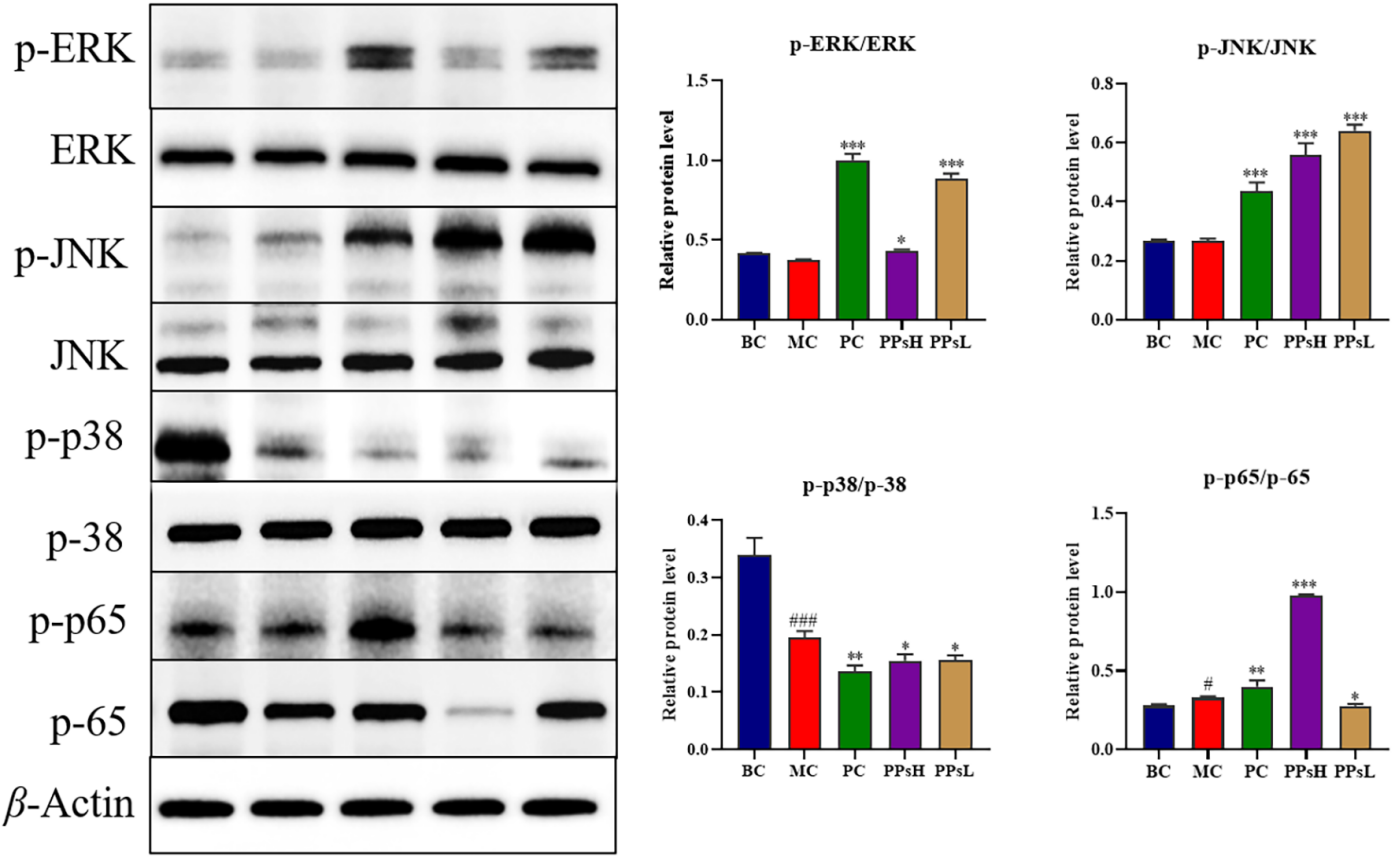
Effect of PPs on proteins expression of NF-κB/MAPK signaling pathways in spleen tissue. ^###^*P* < 0.001 *vs*. BC. ^*^P < 0.05, ^**^P < 0.01, ^***^P < 0.001 *vs*. MC.

The NF-κB/MAPK signaling pathways are signaling cascade mediating immune responses. Western blot analysis of spleen tissue (**Figure 3**) found that CTX inhibited phosphorylation of p38 (*P* < 0.001), p-ERK was lower than that of BC group. Unexpectedly, the phosphorylation levels of JNK showed no significant decrease in macrophages, which may be attributed to the specific time point of sample collection. while PPs treatment increased phosphorylation of ERK, JNK and p65 (*P* < 0.001 or *P* < 0.05). These results suggested that the immunoenhancing effect of PPs might be related to activation of the NF-κB/MAPK signaling pathways.

### 3.4 Effect of PPs on intestinal barrier integrity

**Figure 4:**
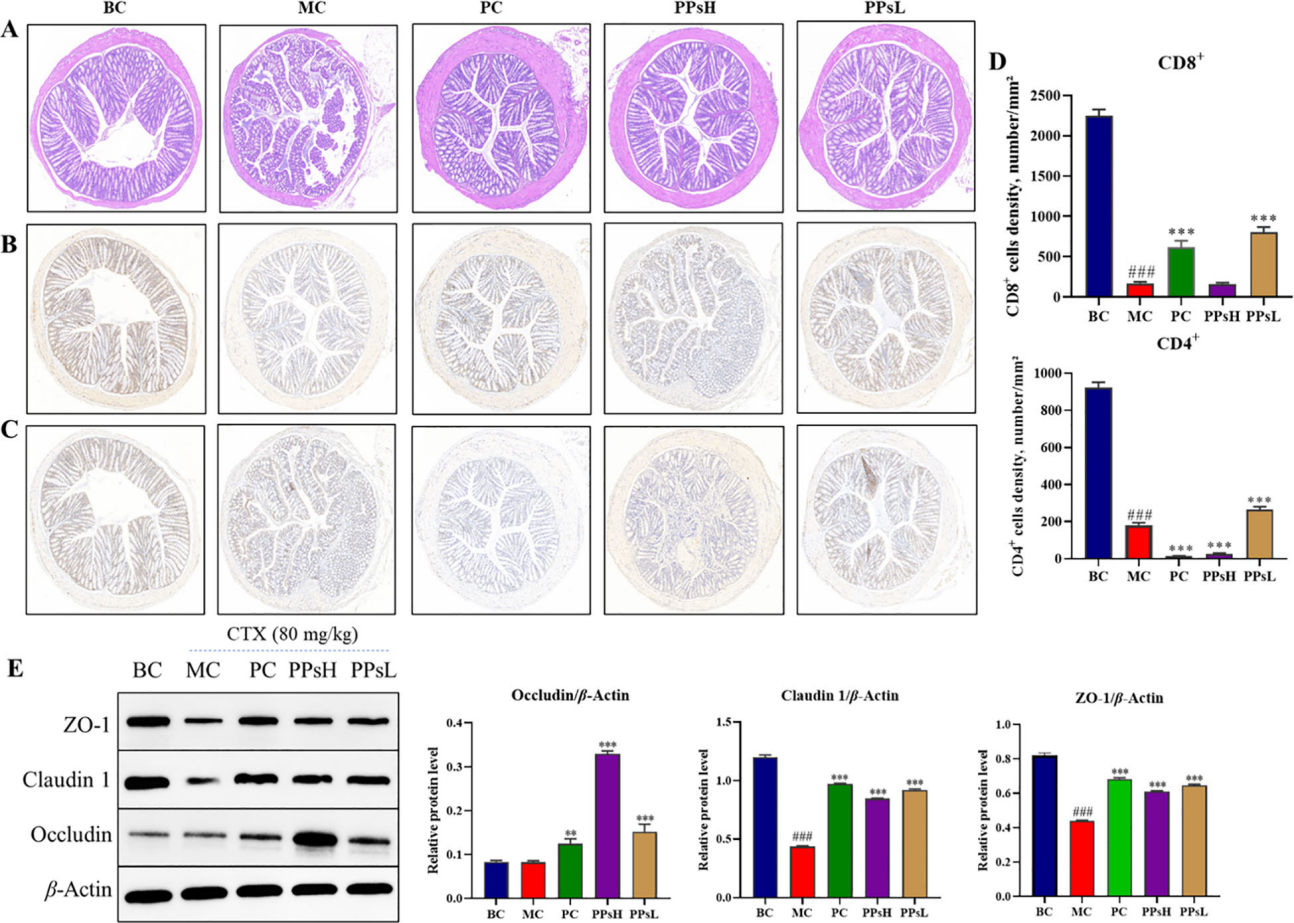
Effect of PPs on intestinal barrier in CTX-induced immunosuppressive mice. **(A)** Pathological observation of small intestinal tissue by H&E staining (×200). **(B)** IHC staining of CD8^+^ T cells (×200). **(C)** IHC staining of CD4^+^ T cells (×200). **(D)** Quantification of CD8^+^ and CD4^+^ T cells. **(E)** Expression of small intestinal tight junction protein. ^###^*P* < 0.001 *vs*. BC. ^**^P < 0.01, ^***^P < 0.001 *vs*. MC.

PPs could enhance the function of the intestinal barrier. Histopathological observation of the small intestine (**Figure 4A**) showed that the BC group exhibited intact mucosal architecture with well-defined villi and crypts. CTX treatment caused clear structural damage, disrupting epithelial integrity and inducing crypt deformation. These morphological changes compromised intestinal function and increased susceptibility to pathogens (19). In contrast, PPs treatment effectively restored the mucosal structure. This restoration indicated an improved capacity for intestinal barrier repair. CD4^+^ and CD8^+^ T lymphocytes play essential roles in adaptive immunity. Immunohistochemical analysis (**Figures 4B–D**) showed that CTX significantly reduced the counts of both CD4^+^ and CD8^+^ T cells (*P* < 0.001). Both PPsH and PPsL restored these cell populations, with the most potent effect observed for PPsL (*P* < 0.001). This suggests that PPs enhance T cell proliferation and activation in the small intestine, thereby supporting local immune responses and cytokine secretion. Western blot analysis of tight junction proteins (**Figure 4E**) revealed that CTX markedly decreased the expressions of Claudin-1, Occludin, and ZO-1. Conversely, PPs treatment significantly upregulated these proteins (*P* < 0.001). The increased expression of these key proteins indicated improved epithelial tightness and reduced intestinal permeability.

### 3.5 Effect of PPs on gut microbiota composition

**Figure 5:**
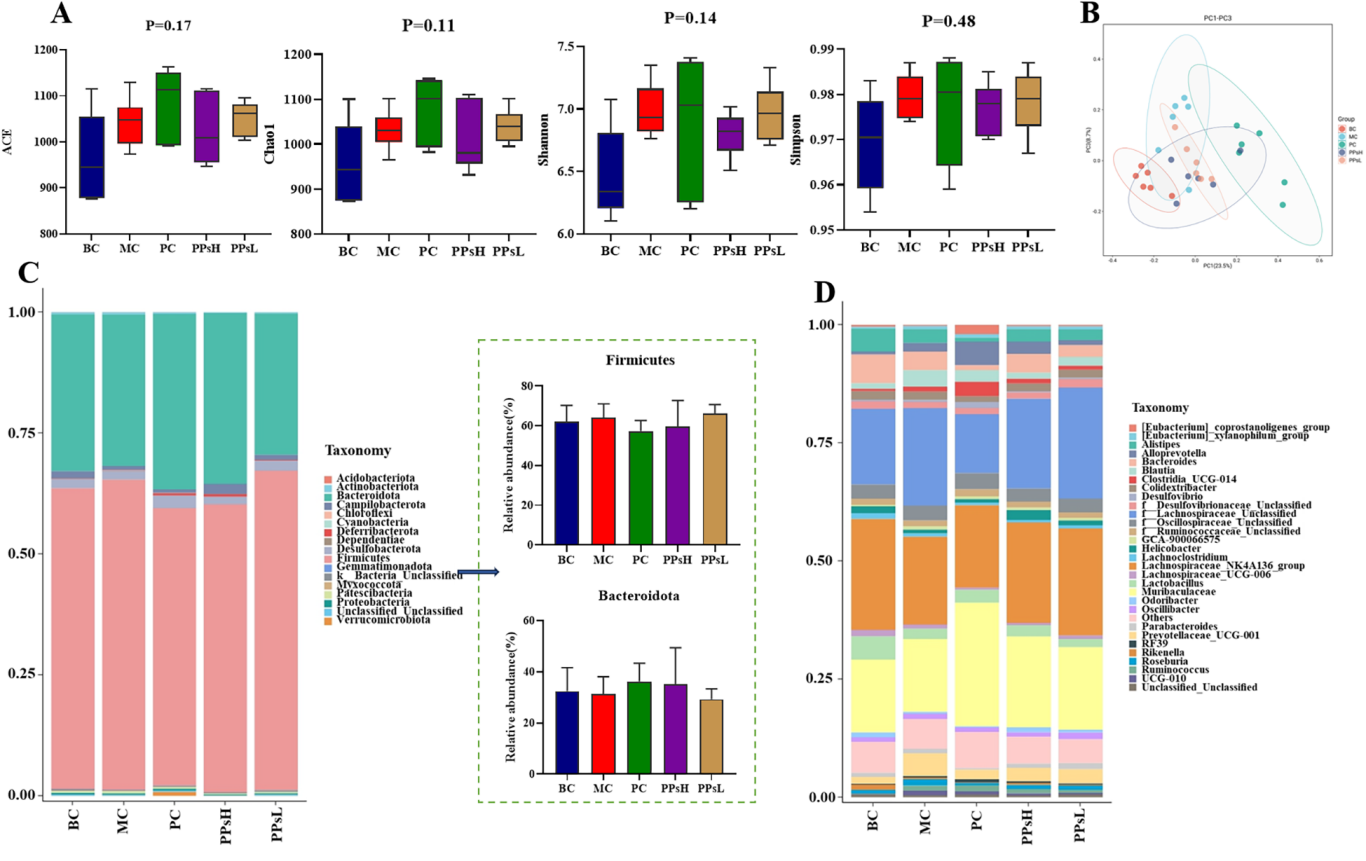
Effect of PPs on gut microbiota in CTX-induced immunosuppressive mice. **(A)** *α-*Diversity indices richness. **(B)** PCoA) on OTU level. **(C)** Gut microbial composition on phylum level. **(D)** Gut microbial composition on genus level.

16S rDNA sequencing revealed a total of 2,818,562 valid reads from 30 cecal content samples across five groups. *α*-Diversity indices (ACE, Chao1, Simpson, and Shannon) showed no significant differences between CTX and PPs groups (*P* > 0.05, **Figure 5A**), indicating that PPs did not alter overall gut microbiota richness. However, *β*-diversity analysis (**Figure 5B**) revealed distinct clustering among groups, with PPsH and PPsL showing clear separation from the CTX group, suggesting compositional shifts in the gut microbiota.

At the phylum level, the microbial community structure was shown in **Figure 5C**. Firmicutes and Bacteroidetes dominated across all groups (> 93% relative abundance), PPs reduced the Firmicutes/Bacteroidetes (F/B) ratio. At the genus level (**Figure 5D**), the relative abundances of Lachnospiraceae_NK4A136_group and *Bacteroides* were higher in the BC group compared to other groups. CTX increased the relative abundances of f:Lachnospiraceae_Unclassified and Prevotellaceae_UCG-001. Compared to the MC group, the PPsH and PPsL groups showed higher relative abundances of *Muribaculaceae* and lower abundances of Prevotellaceae_UCG-001.

Linear Discriminant Analysis Effect Size (LEfSe) analysis identified group-specific biomarker taxa (**Supplementary Figure 1**). Among them, *g_Lactobacillus* was enriched in BC group with a high LDA score, *g_Ruminococcus* in MC group, while *g_Helicobacter* and *g_Oscillibacter* were characteristic PPsH and PPsL groups, respectively. It is noteworthy that a total of 30 bacterial biomarkers were differentially enriched in the PPs-treated groups. These results indicated that under immune-challenged conditions, these gut microbes responded to PPs intervention and collectively participated in modulating the host’s immune response.

### 3.6 Effect of PPs on serum metabolites

**Figure 6:**
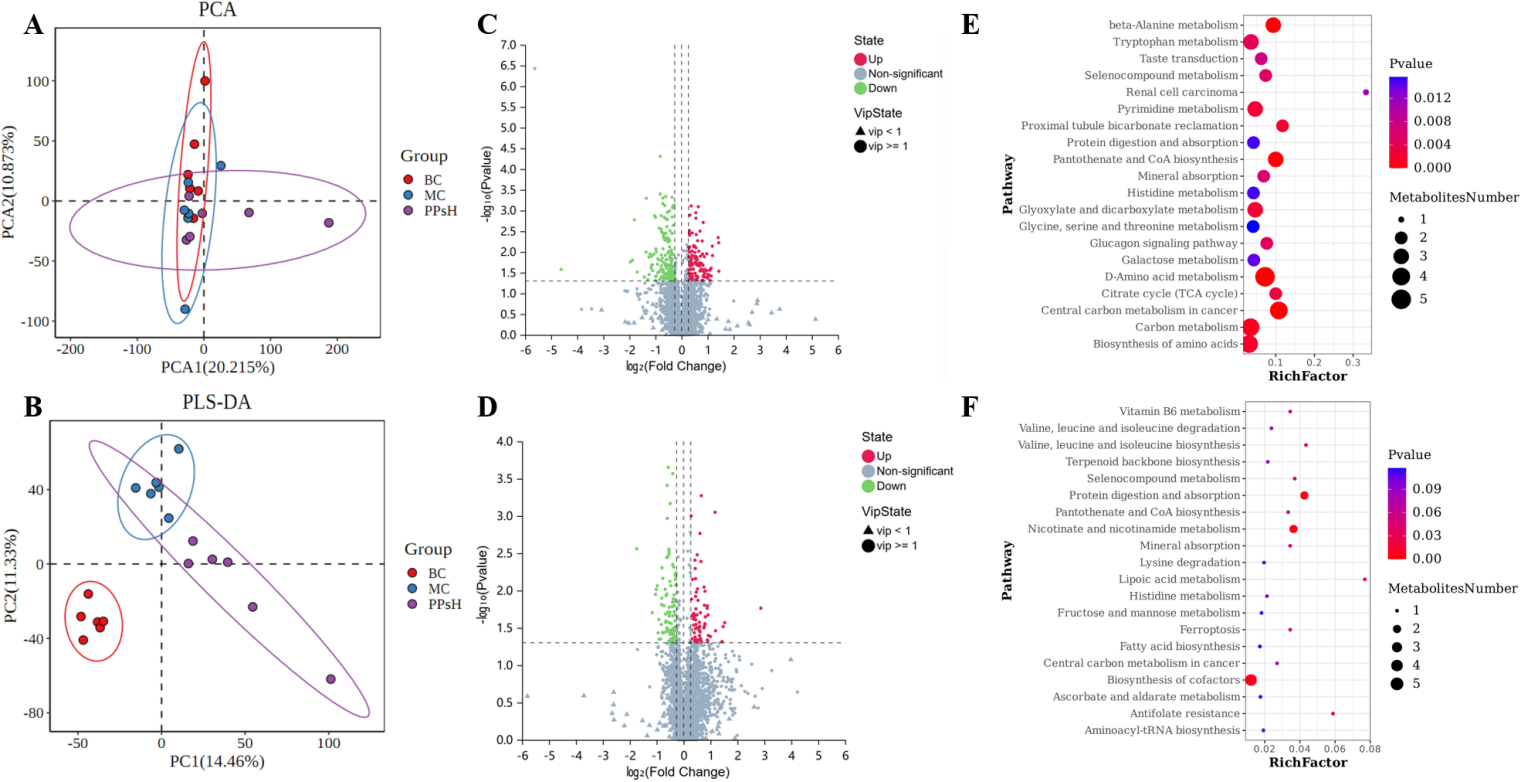
Effect of PPs on serum metabolic parameters in CTX-induced immunosuppressive mice. **(A)** PCA score plot three groups. **(B)** PLS-DA score plot three groups. **(C)** Volcano plot of differential metabolites between BC and MC. **(D)** Volcano plot of differential metabolites between MC and PPsH. **(E)** KEGG pathway enrichment analysis between BC and MC. **(F)** KEGG pathway enrichment analysis between MC and PPsH.

Dietary polysaccharides serve as a major nutrient source for gut bacteria and play a key role in immune regulation, while metabolites act as critical mediators between the host and the microbiota. In previous studies, we investigated changes in the gut microbiome and metabolome. It has been demonstrated that supplementation with PPs altered metabolite profiles. To further investigate the systemic effects, we analyzed serum metabolites after PPs administration. This analysis was performed using a UPLC-Q-Exactive Orbitrap MS system. A total of 3,204 ion features were identified in both positive and negative ion modes. PCA and PLS-DA analyses revealed distinct separations among groups (**Figures 6A, B**). This indicated that CTX markedly altered the serum metabolic profile. Notably, PPs treatment partially reversed these CTX-induced metabolic changes.

Differential metabolites were screened according to predefined criteria, and the results are presented in **Figures 6C, D**. KEGG enrichment analysis was performed to identify affected metabolic pathways (**Figures 6E, F**). Based on the criteria of P < 0.05 and Rich Factor > 0.01, CTX was found to significantly affect 25 pathways (the top 20 are displayed). These included pathways such as D-amino acid metabolism, pantothenate and CoA biosynthesis, carbon metabolism, amino acid biosynthesis, glyoxylate and dicarboxylate metabolism, histidine metabolism, and protein digestion and absorption. PPs treatment, in contrast, significantly modulated six key metabolic pathways. These primarily involved protein digestion and absorption, cofactor biosynthesis, nicotinate and nicotinamide metabolism, lipoic acid metabolism, antifolate resistance, and branched-chain amino acid biosynthesis. Notably, when comparing the PPsH group to the MC group, only the protein digestion and absorption pathway showed a significant reversal, while the other pathways altered by CTX remained unchanged. Similarly, aside from protein digestion and absorption, none of these pathways were significantly altered between the MC and BC groups. These results suggested that PPs ameliorated CTX-induced metabolic disturbances via modulating these key pathways. Among them, the protein digestion and absorption pathway appeared to hold particular potential for alleviating the metabolic disorders caused by CTX.

### 3.7 Correlation analysis of immune markers with gut microbiota and metabolites

**Figure 7:**
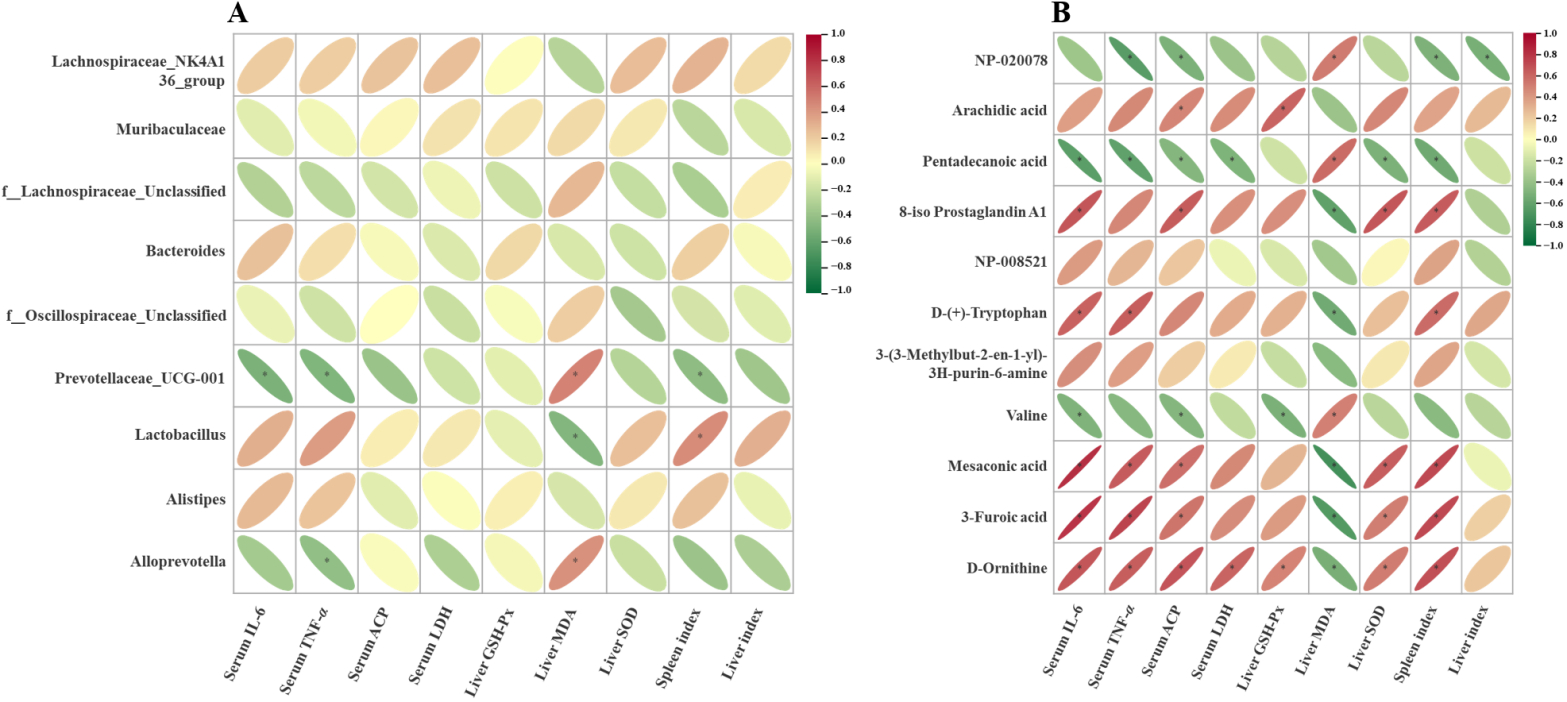
Correlation analysis of biomarkers with gut microbiota **(A)** and metabolites **(B)** *P < 0.05.

To elucidate the mechanism of PPs-mediated immune modulation, we analyzed the correlations among gut microbiota, serum metabolites, and immunological parameters. As shown in **Figure 7A**, the relative abundances of f:Lachnospiraceae_Unclassified, Prevotellaceae_UCG-001, and *Allopretrotella* were negatively correlated with immune and antioxidant markers, including IL-6, TNF-*α*, ACP, LDH, GSH-Px, SOD and spleen index. Conversely, their abundances were positively correlated with MDA. On the contrary, Lachnospiraceae_NK4A136_group and *Lactobacillus* were positively correlated with IL-6, TNF-*α*, ACP, LDH, SOD, spleen index and liver index, whereas negatively correlated with MDA. Similarly, correlation analysis of the main differential metabolites and immunosuppression markers (**Figure 7B**) showed that the contents of arachidic acid, 8-iso prostaglandin A1, D-(+)-tryptophan, mesaconic acid, 3-furoic acid and D-ornithine were positively correlated with IL-6, TNF-*α*, ACP, LDH, GSH-Px, SOD and spleen index, but negatively correlated with MDA. In contrast, metabolites such as NP-020078, pentadecanoic acid and valine showed opposite trends. These findings suggested that PPs modulated gut microbial structure and serum metabolites, jointly contributing to the alleviation of immunosuppression.

## 4 Discussion

The immunomodulatory effects and underlying mechanisms of PPs were systematically investigated in a CTX-induced immunosuppressive mouse model. The findings collectively indicated that PPs could significantly ameliorate inflammation, counteract oxidative stress, restore intestinal barrier integrity, reverse gut microbiota dysbiosis, and modulate host metabolism. These multi-omics findings delineated a clear mechanism of action for PPs. PPs exerted their immunomodulatory effects through regulation of “gut microbiota-host metabolism-immune signaling” axis.

As an effective immunosuppressant and chemotherapeutic agent, CTX impairs immune function by inducing DNA damage and inhibiting the proliferation and differentiation of immune cells such as macrophages (20). Intraperitoneal injection of CTX induced immunosuppression in mice, as evidenced by decreased body weight and decreased immune organ indices. These parameters are key indicators for assessing nonspecific immune competence (21). The spleen, as a representative immune organ, and the liver, as a central metabolic organ, jointly reflect the host’s immune physiological state (22, 23). Our results showed that PPs effectively mitigated key signs of CTX-induced immunosuppression, including weight loss, spleen atrophy, and immune organ damage. Consistent with these findings, PPs elevated serum levels of pivotal immunoregulatory cytokines, such as IL-6 and TNF-*α*, which were critical for activating innate immune responses and coordinating adaptive immunity (24). Together, these immunoenhancing effects indicated PPs enhance both specific (cellular and humoral) and non-specific host immune responses, restoring immune balance disrupted by CTX.

Cytokine secretion is tightly regulated by intracellular signaling cascades, among which NF-κB and MAPK play pivotal roles in immune modulation. Upon immune stimulation, the NF-κB pathway promotes the transcription of key inflammatory factors such as TNF-*α* and IL-6. Concurrently, the MAPK pathways fine-tune the cellular response by activating specific transcription factors that regulate gene expression (25). Excessive activation can trigger cytokine overproduction, whereas balanced activation is essential for immune restoration. Western blot results confirmed that PPs activated both NF-κB and MAPK pathways in the spleen. This observation aligns with previous findings on plant-derived polysaccharides (26, 27), supporting the hypothesis that PPs act as immunomodulators via the coordinated regulation of these signaling axes.

As key effector cells in the innate immune system, macrophages can be functionally characterized by specific enzymatic activities. ACP and LDH are key enzymes indicative of cellular metabolism and immune function. ACP, a lysosomal marker, directly reflects the activation level of macrophages, while LDH, a glycolytic enzyme, reflects cellular energy metabolism. Suppression of these enzymes often indicates a failure in energy metabolism of key immune cells, resulting in compromised immunity (28). PPs administration markedly increased both ACP and LDH activities in the immunocompromised mice, indicating enhanced macrophage activation and restored immune metabolism.

Furthermore, CTX-induced immunosuppression was accompanied by severe oxidative stress due to excessive ROS generation, leading to lipid peroxidation and diminished antioxidant enzyme activities. The resulting oxidative imbalance further exacerbates immune dysfunction. SOD catalyzes the conversion of superoxide radicals, whereas GSH-Px detoxifies peroxides and hydroxyl radicals, both crucial for cellular protection (29, 30). PPs treatment increased hepatic SOD and GSH-Px activities and decreased MDA levels, demonstrating their antioxidant efficacy. This finding indicated that PPs mitigated CTX-induced oxidative damage, partially accounting for their immune-enhancing effects, consistent with previous studies (31).

The intestine not only serves digestive and absorptive functions but also acts as a crucial immune organ through the mucosal immune system (32). The intestinal barrier is critical for maintaining immune homeostasis by preventing the translocation of pathogens and endotoxins (33). CTX disrupts this defense, leading to epithelial damage, crypt deformation, and compromised mucosal immunity (34, 35). Our histological results confirmed that PPs repaired CTX-induced intestinal injury, restoring villus structure and epithelial integrity. This protective effect is comparable to that of other bioactive polysaccharides, such as those derived from *Cordyceps sinensis*, known to enhance villus height and crypt depth (36). Intestinal T lymphocytes, particularly CD4^+^ and CD8^+^ subsets, form the first line of mucosal defense and are essential for maintaining intestinal immune balance (37). CTX decreased their abundance, contributing to local immunosuppression. PPs treatment significantly increased CD4^+^ and CD8^+^ T cell numbers, suggesting an enhanced active role in intestinal immune regulation. Furthermore, Western blot analysis showed that PPs upregulated tight junction proteins (Claudin-1, Occludin, ZO-1), indicating improved epithelial connectivity, reduced permeability, and prevention of pathogen translocation (38). These effects collectively demonstrated that PPs reinforced intestinal barrier integrity both structurally and immunologically.

The gut microbiota plays a central role in maintaining intestinal barrier homeostasis and systemic immunity through interactions with epithelial and immune cells (39). The gut microbiota modulates host endocrine and neural signaling and produces beneficial metabolites, such as short-chain fatty acids. Through these activities, it engages in dynamic crosstalk with the host immune system. This bidirectional communication is essential for jointly sustaining immune homeostasis (40). Dysbiosis, characterized by decreased microbial diversity or imbalance between beneficial and pathogenic species, has been closely linked to immune dysfunction (41). Polysaccharides are poorly digested in the upper gastrointestinal tract but undergo fermentation by intestinal microbiota, leading to the enrichment of beneficial bacterial populations and production of immunoregulatory metabolites (42, 43). Integrated 16S rDNA sequencing and metabolomics analyses revealed that PPs intervention reshaped the gut microbiota and concurrently corrected serum metabolic disorders induced by CTX. Specifically, PPs increased the relative abundances of Lachnospiraceae_NK4A136_group and *Lactobacillus*, while decreasing those of Prevotellaceae_UCG-001, f:Lachnospiraceae_Unclassified, and *Alloprevotella*. Notably, Lachnospiraceae_NK4A136_group, a butyrate-producing bacterium, showed a positive correlation with immune organ indices and immunoglobulin levels (IgG, IgM) (44). *Lactobacillus*, a well-known probiotic genus, supports immune balance by producing lactic acid to lower intestinal pH, inhibiting pathogen colonization, promoting regulatory T cell differentiation, and strengthening epithelial junctions (45). Concurrently, PPs administration significantly modulated key serum metabolic pathways, including those related to protein digestion and absorption, nicotinate and nicotinamide metabolism, lipoic acid metabolism, and branched-chain amino acid biosynthesis. These coordinated changes collectively demonstrate that PPs exert immunomodulatory effects by restoring homeostasis along the gut microbiota-host metabolism axis. Correlation analysis further indicated close associations among these metabolites, beneficial bacterial taxa, and immune/antioxidant markers. Collectively, our findings indicated that PPs likely function as prebiotics. They remodeled the composition of the gut microbiota and the host’s metabolic landscape. These changes ultimately enhanced systemic immune resilience and mitigated the immunosuppressive effects induced by CTX.

## 5 Conclusions

In conclusion, PPs effectively ameliorated CTX-induced immunosuppression in mice by enhancing immune responses, preserving intestinal integrity, and restoring gut microbiota-metabolite balance. Mechanistically, PPs promoted cytokine secretion via modulation of protein expression in the NF-κB and MAPK signaling pathways. Concurrently, they increased intestinal microbial richness and reshaped the microbial community, enriching beneficial taxa while suppressing pathogenic ones, thereby alleviating microbial dysbiosis. Furthermore, PPs strengthened the intestinal barrier by upregulating tight junction proteins and increasing the population of intestinal CD4^+^ and CD8^+^ T lymphocytes, contributing to improved mucosal immunity. The correction of metabolic disturbances further supports their systemic immunoprotective effects. Collectively, these findings highlight PPs as promising natural immunomodulators and potential functional ingredients for mitigating CTX-induced immunosuppression.

## Data Availability

The original contributions presented in the study are included in the article/**Supplementary Material**. Further inquiries can be directed to the corresponding author.
