# Supplementary figures and images for: Polysaccharides from Psoralea corylifolia alleviate CTX-induced immunosuppression in mice by modulating gut microbiota- metabolite-immune signaling
Source: Front Immunol. 2026 Mar 3;17:1759122. doi: 10.3389/fimmu.2026.1759122 (PMC12992029; doi:10.3389/fimmu.2026.1759122)

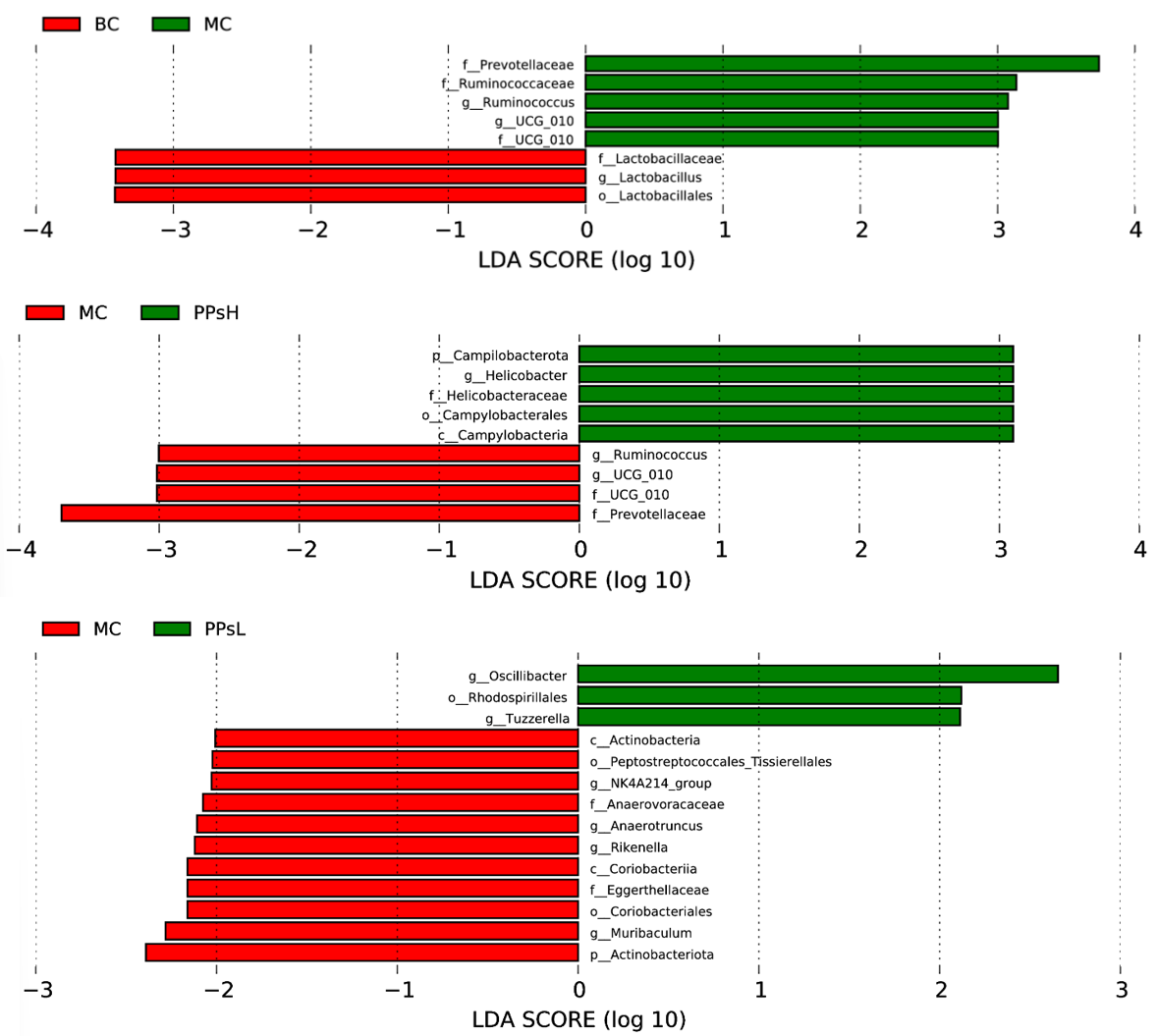

Supplement: Supplementary file 1 [file Image1.tif]
